# Association between malaria exposure and Kaposi's sarcoma-associated herpes virus seropositivity in Uganda

**DOI:** 10.1111/tmi.12464

**Published:** 2015-02-09

**Authors:** Angela Nalwoga, Stephen Cose, Katie Wakeham, Wendell Miley, Juliet Ndibazza, Christopher Drakeley, Alison Elliott, Denise Whitby, Robert Newton

**Affiliations:** 1 Medical Research Council/Uganda Virus Research InstituteEntebbe, Uganda; 2 London School of Hygiene & Tropical MedicineLondon, UK; 3 Institute of Cancer Sciences, University of GlasgowGlasgow, UK; 4 Viral Oncology Section, Frederick National Laboratory for Cancer ResearchFrederick, MD, USA; 5 University of YorkYork, UK; 6 International Agency for Research on CancerLyon, France

**Keywords:** Kaposi's sarcoma-associated herpes virus, malaria, Uganda

## Abstract

**Objective:**

Unlike other herpes viruses, Kaposi's sarcoma-associated herpes virus (KSHV) is not ubiquitous worldwide and is most prevalent in sub-Saharan Africa. The reasons for this are unclear. As part of a wider investigation of factors that facilitate transmission in Uganda, a high prevalence country, we examined the association between antimalaria antibodies and seropositivity against KSHV.

**Methods:**

Antibodies against *P. falciparum* merozoite surface protein (*Pf*MSP)-1, *P. falciparum* apical membrane antigen (*Pf*AMA)-1 and KSHV antigens (ORF73 and K8.1) were measured in samples from 1164 mothers and 1227 children.

**Results:**

Kaposi's sarcoma-associated herpes virus seroprevalence was 69% among mothers and 15% children. Among mothers, KSHV seroprevalence increased with malaria antibody titres: from 60% to 82% and from 54% to 77%, comparing those with the lowest and highest titres for *Pf*MSP-1 and *Pf*AMA-1, respectively (*P* < 0.0001). Among children, only antibodies to *Pf*AMA-1 were significantly associated with KSHV seropositivity, (*P* < 0.0001). In both mothers and children, anti-ORF73 antibodies were more strongly associated with malaria antibodies than anti-K8.1 antibodies.

**Conclusion:**

The association between malaria exposure and KSHV seropositivity suggests that malaria is a cofactor for KSHV infection or reactivation.

## Introduction

Kaposi's sarcoma-associated herpes virus (KSHV) is the causative agent of Kaposi's sarcoma (KS) [1,2]. In general, the distribution of KSHV mirrors the distribution of KS [3]. Unlike other herpes viruses that are ubiquitous in human populations, KSHV and KS demonstrate substantial geographical variation worldwide [4]. The prevalence of KSHV is highest in sub-Saharan Africa, followed by the Mediterranean countries, and prevalence is lowest in Northern European and Asian countries [5]. The principal modes of transmission of KSHV may also differ between geographical locations [6]. In sub-Saharan Africa, KSHV transmission occurs through saliva throughout life, but particularly in childhood [3]. In low prevalence settings transmission mainly occurs later in life, perhaps through sexual contact [3].

As all other known human herpes viruses are ubiquitous worldwide, the geographic variation in prevalence of KSHV is anomalous and the factors that sustain higher rates of transmission in sub-Saharan Africa than elsewhere are unclear. In a cross-sectional study of women, hookworm infection, *Mansonella perstans* and malaria parasitaemia were independently associated with KSHV seropositivity; in children, only malaria parasitaemia showed an association with KSHV seropositivity, but prevalence of the other parasites was very low [7,8]. Helminths skew the immune response to a Th2 response and cause immunosuppression [9,10]; this immunosuppression could lead to loss of viral control and could consequently cause viral replication. Malaria infection impairs the T-cell immune response and causes polyclonal activation of B cells [11,12]. Both malaria and helminth infections could lead to KSHV reactivation in co-infected individuals.

Repeated malaria exposure has detrimental effects on immune function [13–16]. These could lead to loss of immune surveillance of KSHV latently infected cells, consequently causing viral reactivation and replication. The effect of intense malaria exposure on EBV reactivation (a related gamma herpesvirus) has been investigated, and it was shown that exposure to malaria facilitates EBV transmission [17]. Individuals in areas with high malaria transmission in Kenya were more likely to be EBV seropositive and were at higher risk of Burkitt's lymphoma than individuals in areas with low malaria transmission in Kenya [17,18]. Together, this suggests that malaria impacts not just transmission of EBV, but also the immune response to infection; the same may be true in relation to KSHV and KS. The purpose of this study was to investigate the effect of malaria exposure, determined by measurement of antimalaria antibodies, on KSHV seropositivity in Ugandan mothers and their children.

## Methods

### Study design and population

This was a cross-sectional study carried out within the context of a clinical trial, the Entebbe Mother and Baby study (EMaBS) (ISRCTN32849447). EMaBS is an ongoing birth cohort that originated as a double-blind, randomised placebo-controlled trial designed to determine the impact of helminth infections and their treatment on vaccine responses and infectious diseases outcomes; the details have been reported elsewhere [19,20]. A total of 2507 pregnant women from Entebbe, Uganda, who consented, were recruited into EMaBS and they have been followed, with their children, for 10 years.

### Ethical approval

This study was approved by the Science and Ethics Committee (SEC) of the Uganda Virus Research Institute, Uganda National Council for Science and Technology and the London School of Hygiene & Tropical Medicine Research Ethics Committee.

### KSHV Serology

Stored plasma samples taken from 1164 mothers in the early post-partum period, and from 1227 of their 5-year old children, were screened for the presence of KSHV antibodies using an enzyme-linked immunosorbent assay (ELISA) for recombinant proteins to a lytic structural glycoprotein, K8.1 and a latent nuclear protein latency-associated nuclear antigen (LANA) encoded by ORF73. Each plate contained three positive and three negative controls. Each assay cut-off was calculated based on the performance of the negative controls. This procedure has been reported elsewhere [21,22].

### Malaria serology

The same plasma samples were tested for malaria antibodies using two *P. falciparum* antigens: merozoite surface protein (MSP)-1 and apical membrane antigen (AMA)-1 [23]. A pool of malaria positive plasma samples from patients known to be infected with malaria was used to make standard dilutions. This pool was diluted serially five times starting from 1:50 for MSP-1 and 1:100 for AMA-1 to make six standards with a fourfold dilution increment. Optical densities (ODs) obtained were then exported into Microsoft Excel, and antibody titres for each sample and each antigen were derived from the standard curve, of ODs. Blank wells were used to subtract background absorbance from the standards and the samples. This procedure has been reported elsewhere [24].

### Statistical analysis

Statistical analysis was performed using Stata-12 software (STATA® 12.1, Statacorp, College Station, USA). Separately for mothers and children, odds ratios (ORs) and 95% confidence intervals (CIs) were calculated using the Mantel–Haenszel test and logistic regression to obtain crude and adjusted odds ratios and *P*-values; all *P*-values are two-sided. To determine the effect of malaria antibodies on KSHV seropositivity, we grouped malaria antibody titres into three groups, for mothers and children separately: low, middle and high. The selection of the groups was not based on any biological or clinical criteria but on the frequency distribution of the titres. Antibody titres from below the 25th quartile were grouped as low, from the 25th to 75th quartile as middle and above the 75th quartile as high. As grouping continuous risk factors leads to loss of information [25], we also analysed the effect of malaria antibody titres as a continuous variable.

## Results

To determine the level of malaria exposure, we quantified antibody titres to *Plasmodium falciparum* malaria using two antigens, *Pf*MSP-1 and *Pf*AMA-1. As expected, antibody titres to both *Pf*MSP-1 and *Pf*AMA-1 were much higher in mothers compared to children. The median antibody titres to *Pf*MSP-1 and *Pf*AMA-1 among mothers were 737.5 and 600.2, respectively, while the median antibody titres to *Pf*MSP-1 and *Pf*AMA-1 among children were 103.4 and 17.2, respectively. To establish the seroprevalence of KSHV among mothers and their children, we tested for KSHV-specific antibodies using two KSHV antigens, K8.1 and ORF73. Individuals with antibodies to either ORF73 or K8.1 were considered to be KSHV seropositive, and individuals with antibodies to neither ORF73 nor K8.1 were considered to be KSHV seronegative. The seroprevalence of KSHV was 69% (806/1164) among mothers and 15% (185/1227) among children. Among mothers, 64% (744) were positive for anti-ORF73 antibodies and 46% (539) were positive for anti-K8.1 antibodies. Among children, 8% (96) were positive for anti-ORF73 antibodies and 12% (148) were positive for anti-K8.1 antibodies.

Tables 1 and 2 show crude and adjusted associations between KSHV seropositivity, socio-demographic factors and other clinical factors among mothers and children, respectively. Household socioeconomic status and location were independently associated with KSHV seropositivity both in the mothers and in the children [7,8]. Age was crudely associated with KSHV seropositivity (*P* = 0.01) among mothers, but the association was lost when we adjusted for household socio-economic status and location. Because all children were 5 years old, age was not included in the analysis of children's data.

**Table 1:** Prevalence of KSHV among women. Crude and adjusted associations with KSHV serostatus and socio-demographics and some clinical factors among 1164 mothers

Risk factor	Prevalence of KSHV among women	Crude odds ratio (95% CI)	*P*	Adjusted odds ratios (95% CI)	*P*
Age group					
14–19	75% (212/282)	1	**0.01** (trend)	1	0.07 (trend)
20–24	69% (299/433)	0.74 (0.53–1.03)		0.75 (0.53–1.06)	
25–29	66% (175/265)	0.64 (0.44–0.93)		0.70 (0.47–1.02)	
30+	65% (120/184)	0.62 (0.41–0.93)		0.68 (0.44–1.03)	
Household SES					
1 (lowest)	82% (49/60)	1	**<0.0001** (trend)	1	**<0.0001** (trend)
2	75% (77/103)	0.66 (0.30–1.47)		0.70 (0.31–1.55)	
3	72% (254/350)	0.59 (0.30–1.19)		0.56 (0.78–1.13)	
4	68% (228/335)	0.48 (0.24–0.96)		0.46 (0.23–0.93)	
5	65% (145/224)	0.41 (0.20–0.84)		0.43 (0.21–0.88)	
6 (highest)	49% (33/67)	0.22 (0.10–0.49)		0.22 (0.10–0.49)	
Location					
Urban*	65% (286/439)	1	**0.002** (trend)	1	**0.002** (trend)
Peri-urban†	70% (309/444)	1.22 (0.92–1.62)		1.26 (0.94–1.69)	
Rural‡	76% (206/271)	1.70 (1.21–2.38)		1.77 (1.24–2.52)	
Tribe					
Buganda	70% (419/601)	1	0.7		
Other tribes§	69% (386/562)	0.95 (0.74–1.22)			
HIV seronegative	69% (694/1004)	1	0.8		
HIV seropositive	70% (112/160)	1.04 (0.72–1.50)			
Anaemia					
No	67% (472/699)	1	0.1		
Yes	72% (334/465)	1.23 (0.95–1.59)			

SES (socio-economic status), CI (confidence interval). KSHV seropositive is defined as seropositive to either ORF73, and/or K8.1. A composite variable for household socio-economic status was derived based on home building materials, number of room and items collectively owned.

Adjusted odds ratios were adjusted for age group, household socio-economic status and location.

* Urban is Entebbe area.

† Peri-urban are Kigungu and Manyago areas.

‡ Rural are Katabi road side and Katabi far from the road areas.

§ Other tribes include Banyankole, Batoro, Basoga, Luo, Banyarwanda and any other tribes.

Bold values are statistically significant

**Table 2:** Prevalence of KSHV among five-year-old children. Crude and adjusted associations with KSHV serostatus and socio-demographic factors among 1227 children

Risk factor	Prevalence of KSHV among children	Crude odds ratio (95% CI)	*P*	Adjusted odds ratios (95% CI)	*P*
Household SES					
1 (lowest)	23% (16/71)	1	**0.05** (trend)	1	**0.04** (trend)
2	18% (14/81)	0.72 (0.32–1.60)		0.75 (0.33–1.68)	
3	16% (60/368)	0.67 (0.36–1.25)		0.65 (0.35–1.22)	
4	12% (43/352)	0.48 (0.25–0.91)		0.46 (0.24–0.88)	
5	15% (38/255)	0.61 (0.31–1.16)		0.57 (0.30–1.11)	
6 (highest)	12% (9/78)	0.45 (0.18–1.09)		0.44 (0.18–1.08)	
Location					
Urban*	14% (64/463)	1	**0.01** (trend)	1	**0.01** (trend)
Peri-Urban†	13% (65/498)	0.94 (0.65–1.36)		0.88 (0.6–1.29)	
Rural‡	22% (56/255)	1.75 (1.18–2.61)		1.80 (1.20–2.69)	
Maternal age group					
14–19	13% (33/253)	1	0.3 (trend)		
20–24	16% (76/473)	1.28 (0.82–1.98)			
25–29	13% (38/294)	0.99 (0.60–1.63)			
30+	19% (38/207)	1.50 (0.90–2.49)			
Sex					
Boys	16% (101/626)	1	0.3		
Girls	14% (84/601)	0.84 (0.62–1.15)			
Tribe					
Buganda	15% (103/683)	1	0.9		
Other tribes§	15% (82/544)	0.99 (0.73–1.37)			

SES (socio-economic status), CI (confidence interval) KSHV seropositive is defined as seropositive to either ORF73 and/or K8.1. A composite variable for household socio-economic status was derived based on home building materials, number of room and items collectively owned.

Adjusted odds ratios adjusted for household socio-economic status and location.

* Urban is Entebbe area.

† Peri-urban are Kigungu and Manyago areas.

‡ Rural are Katabi road side and Katabi far from the road areas.

§ Other tribes include Banyankole, Batoro, Basoga, Luo, Banyarwanda and any other tribe. Bold values are statistically significant

Among mothers, malaria antibodies to both *Pf*MSP-1and *Pf*AMA-1 were strongly associated with KSHV seropositivity. The odds of being KSHV seropositive among mothers with high malaria antibody titres, compared to those with low malaria antibody titres, were 2.67 (*P* < 0.0001) and 2.43 (*P* < 0.0001) for *Pf*MSP-1 and *Pf*AMA-1 antibodies, respectively (Table 3). Among children, malaria antibodies to *Pf*AMA-1 were strongly associated with KSHV seropositivity. The odds of being KSHV seropositive among children with high *Pf*AMA-1 antibody titres, compared to those with low *Pf*AMA-1 antibody titres, were 1.59 (*P* = 0.02) (Table 4). *Pf*MSP-1 antibody titres were not significantly associated with KSHV seropositivity among children (Table 4).

**Table 3:** Association of antimalaria antibodies with prevalence of KSHV among women. Crude and adjusted associations between KSHV serostatus and malaria antibody titres among 1164 mothers

Risk factor	Prevalence of KSHV among women	Crude odds ratio (95% CI)	*P*	Adjusted odds ratio (95%CI)	P*
*Pf*MSP-1 titres					
Lowest	60% (174/292)	1	**<0.0001**	1	**<0.0001**
Middle	68% (394/581)	1.43 (1.07–1.91)		1.47 (1.07–2.03)	
Highest	82% (238/291)	3.05 (2.09–4.45)		2.67 (1.77–4.04)	
*Pf*AMA-1 titres					
Lowest	54% (158/293)	1	**<0.0001**	1	**<0.0001**
Middle	73% (423/580)	2.30 (1.72–3.09)		2.08 (1.5–2.87)	
Highest	77% (225/291)	2.91 (2.04–4.17)		2.43 (1.63–3.62)	

* Adjusted odds ratios were adjusted for hookworm infection, *Mansonella perstans* infection, socio-demographic factors (age group, household socio-economic status, location) and HIV status. *Pf*MSP-1 (*Plasmodium falciparum* merozoite surface protein-1), *Pf*AMA-1 (*Plasmodium falciparum* apical membrane antigen-1), CI (confidence interval) *Pf*MSP-1 and *Pf*AMA-1 antibody titres were analysed using separate regression models. KSHV seropositive is defined as seropositive to either ORF73 and/or K8.1.Bold values are statistically significant

**Table 4:** Association of antimalaria antibodies with prevalence of KSHV among children. Crude and adjusted associations between KSHV serostatus and malaria antibody titres among 1227 children

Risk factor	Prevalence of KSHV among children	Crude odds ratios (95% CI)	*P*	Adjusted odds ratio (95% CI)	P*
*Pf*MSP-1 titres					
Lowest	14% (42/307)	1	0.2	1	0.4
Middle	15% (89/614)	1.07 (0.72–1.59)		1.05 (0.70–1.56)	
Highest	18% (54/306)	1.35 (0.87–2.10)		1.23 (0.79–1.92)	
*Pf*AMA-1 titres					
Lowest	14% (43/313)	1	**0.002**	1	**0.02**
Middle	12% (73/608)	0.86 (0.57–1.28)		0.84 (0.56–1.26)	
Highest	23% (69/306)	1.83 (1.20–2.78)		1.59 (1.03–2.45)	

* Adjusted odds ratios were adjusted for socio-demographic factors (sex, household socio-economic status and location). *Pf*MSP-1 (*Plasmodium falciparum* merozoite surface protein-1), *Pf*AMA-1 (*Plasmodium falciparum* apical membrane antigen-1) CI (confidence interval). *Pf*MSP-1 and *Pf*AMA-1 antibody titres were analysed using separate regression models. KSHV seropositive is defined as seropositive to either ORF73 and/or K8.1.Bold values are statistically significant

We also analysed antibodies to ORF73 and K8.1 separately. Among mothers, the association between malaria antibodies and the presence of ORF73 seropositivity was stronger than the association between malaria antibodies and K8.1 seropositivity (Table 5). Findings among children were similar (Table 6), although mothers had a stronger association between malaria antibodies and KSHV seropositivity (compare Tables5 and 6).

**Table 5:** Associations between antimalaria antibodies and anti-KSHV antibodies ORF73 and K8.1 among women. Crude and adjusted associations between KSHV antibody responses malaria antibody titres among 1164 mothers

	ORF73					K8.1				
Risk factor	Prevalence of women with anti-ORF73 antibodies	Crude odds ratio (95% CI)	P*	Adjusted odds ratio (95% CI)	P*	Prevalence of women with anti-K8.1 antibodies	Crude odds ratio (95% CI)	*P*	Adjusted odds ratio (95% CI)	P*
*Pf*MSP-1 titres										
Lowest	53% (154/292)	1	**<0.0001**	1	**<0.0001**	42% (122/292)	1	**0.003**	1	**0.01**
Middle	62% (359/581)	1.45 (1.09–1.93)		1.42 (1.04–1.93)		45% (259/581)	1.12 (0.84–1.49)		1.21 (0.89–1.65)	
Highest	79% (231/291)	3.45 (2.39–4.97)		2.99 (2.01–4.45)		54% (158/291)	1.66 (1.19–2.30)		1.57 (1.11–2.24)	
*Pf*AMA-1 titres										
Lowest	45% (132/293)	1	**<0.0001**	1	**<0.0001**	40% (118/293)	1	**0.03**	1	0.1
Middle	69% (399/580)	2.69 (2.01–3.59)		2.47 (1.80–3.38)		48% (277/580)	1.36 (1.02–1.80)		1.33 (0.97–1.80)	
Highest	73% (231/291)	3.33 (2.35–4.71)		3.05 (2.08–4.47)		49% (144/291)	1.45 (1.05–2.02)		1.34 (0.94–1.91)	

* Adjusted odds ratios were adjusted for hookworm infection, *Mansonella perstans* infection, socio-demographic factors (age group, household socio-economic status, location) and HIV status. *Pf*MSP-1 (*Plasmodium falciparum* merozoite surface protein-1), *Pf*AMA-1 (*Plasmodium falciparum* apical membrane antigen-1), CI (confidence interval), *Pf*MSP-1 and *Pf*AMA-1 antibody titres were analysed using separate regression models.Bold values are statistically significant

**Table 6:** Crude and adjusted associations between KSHV antibody responses and malaria antibody titres among 1227 children

	ORF73					K8.1				
Risk factor	Prevalence of children with anti-ORF73 antibodies	Crude odds ratios (95% CI)	*P*	Adjusted odds ratio (95% CI)	P*	Prevalence of children with K8.1 antibodies	Crude odds ratios (95% CI)	*P*	Adjusted odds ratio (95% CI)	P*
*Pf*MSP-1 titres										
Lowest	6% (18/307)	1	0.05	1	0.1	13% (939/307)	1	0.6	1	0.9
Middle	8% (47/614)	1.33 (0.76–2.33)		1.28 (0.72–2.27)		11% (66/614)	0.83 (0.54–1.26)		0.82 (0.53–1.25)	
Highest	10% (31/306)	1.81 (1–3.31)		1.61 (0.87–2.99)		14% (43/306)	1.12 (0.71–1.79)		1 (0.62–1.61)	
*Pf*AMA-1 titres										
Lowest	5% (16/313)	1	**<0.0001**	1	**<0.0001**	12% (36/313)	1	0.05	1	0.2
Middle	5% (33/608)	1.07 (0.58–1.97)		1.01 (0.54–1.88)		10% (61/608)	0.86 (0.55–1.33)		0.84 (0.54–1.31)	
Highest	15% (47/306)	3.37 (1.86–6.08)		2.76 (1.50–5.06)		17% (51/306)	1.54 (0.97–2.44)		1.30 (0.81–2.09)	

* Adjusted odds ratios were adjusted for socio-demographic factors (sex, household socio-economic status and location). *Pf*MSP-1 (*Plasmodium falciparum* merozoite surface protein-1), *Pf*AMA-1 (*Plasmodium falciparum* apical membrane antigen-1), CI (confidence interval), *Pf*MSP-1 and *Pf*AMA-1 antibody titres were analysed using separate regression models.Bold values are statistically significant

Treating antimalaria antibody titres as a continuous variable, among mothers, for every fourfold increase in malaria antibody titre, the odds of being KSHV seropositive increased by 67% (*P* < 0.0001) for *Pf*MSP-1 antibody titres and 60% (*P* < 0.0001) for *Pf*AMA-1 antibody titres. In the children, for every fourfold increase in *Pf*AMA-1 antibody titres, the odds of being KSHV seropositive increased by 53% (*P* < 0.0001). *Pf*MSP-1 antibody titres were not associated with KSHV seropositivity among children.

## Discussion

To our knowledge, this is the first study to investigate malaria antibodies in relation to KSHV seropositivity. Its major findings are as follows: (1) KSHV seropositivity was strongly associated with malaria antibodies to both *Pf*MSP-1 and *Pf*AMA-1 in the mothers. (2) In the children, KSHV seropositivity was highly associated with malaria antibody titres to *Pf*AMA-1 but not *Pf*MSP-1. (3) In both mothers and their children, the association between malaria antibodies and KSHV antibodies was much stronger with ORF73 antibodies than K8.1 antibodies.

Kaposi's sarcoma-associated herpes virus seropositivity was strongly associated with both malaria antibodies in the mothers. In this study, we have used malaria antibodies as a proxy measure of malaria exposure. Continuous exposure to malaria infection leads to the development of an antibody response, some of which is associated with protective immunity to clinical disease [26–28]. The exact correlates of protection for malaria are unknown, although high malaria antibody titres to the malarial antigens used here (MSP-1 and AMA-1) have been associated with protection in some studies [26,29–31]and have, more recently, been used to demonstrate cumulative exposure with age to classify areas of different malaria endemicity [32,33]. Therefore, malaria antibody titres correlate with malaria infection exposure. Repeated exposure to malaria infection has been associated with detrimental effects on immune function [14,16] such as T- and B-cell exhaustion [13] and impairment of myeloid lineage cells such as dendritic cells function [15]. During KSHV latent infection, the immune system, through the action of cytotoxic T lymphocytes, prevents viral replication and hence keeps the virus in latency. The loss of T-cell immunity during repeated malaria infections could lead to loss of viral control and could consequently lead to KSHV transmission as a result of viral replication; (2) and/or render KSHV-negative individuals more susceptible to the infection, hence leading to KSHV acquisition.

The association between KSHV seropositivity and malaria antibodies could also be directly due to the effects of malaria parasite infection. We have previously shown that asymptomatic malaria parasitaemia is associated with KSHV seropositivity [7,8]. Possible mechanisms through which malaria infection could impact KSHV replication are (i) immunosuppression and (ii) polyclonal activation of KSHV latently infected B cells; malaria parasites cause both.

We found that, in both mothers and their children, the association between KSHV antibodies and malaria antibodies is much stronger with ORF73 antibodies than with K8.1 antibodies. ORF73 encodes LANA (latently associated nuclear antigen), which is expressed during latency to facilitate KSHV episomal replication, segregation of the KSHV genomes to daughter cells and viral oncogenicity [34]. K8.1, in contrast, is a structural protein expressed during lytic replication. There are a number of possible mechanisms that may explain this; however, there is considerable heterogeneity in the antibody response to more than 85 KSHV antigens [22]. Further studies are needed to understand the interaction of KSHV antigens with the immune system.

Unlike in the mothers, we observed that KSHV seropositivity is associated with antibodies to *Pf*AMA-1 but not *Pf*MSP-1 in the children. Possible explanations (either alone or in combination) could be that (i) the KSHV seroprevalence was very low in children (15%); this might have hindered detection of significant associations. (ii) The *Pf*AMA-1 used is a larger molecule, will have more epitopes and therefore, is potentially a more sensitive biomarker for exposure [35]. Also, antibodies to *Pf*MSP-1 take longer to develop, and hence their levels during early life are less well correlated with malaria exposure [29].

Our sample sizes were large enough to allow detection of statistically significant associations, at least in the mothers. Also, data on possible confounders were available; therefore, we were able to adjust for them. We were also able to detect specific antibody responses to more than one antigen per infection. Potential weaknesses were the lack of any information on the temporality, which could have effects on malaria infection distribution, and the cross-sectional study design, which did not allow us to ascertain when malaria antibody titres increased and when KSHV primary infection occurred.

Repeated exposure to malaria infection has detrimental effects on immune function, for example by leading to B- and T-cell exhaustion. We have shown that exposure to malaria infection, detected using malaria antibodies, is highly associated with KSHV seropositivity. Therefore, it is possible that malaria exposure is facilitating reactivation of KSHV, and perhaps also increasing the susceptibility to infection, which in turn may lead to increased transmission.
